# A Case of Neurosarcoidosis-Induced Syndrome of Inappropriate Secretion of Antidiuretic Hormone Diagnosed with Neuroendoscopy

**DOI:** 10.1155/2018/9496149

**Published:** 2018-08-06

**Authors:** Shiko Gen, Akio Ogawa, Koji Kanai, Kanako Nobe, Naofumi Ikeda, Atsuko Mochizuki, Kazuo Tokushige

**Affiliations:** ^1^Department of Nephrology, Saitama Sekishinkai Hospital, Saitama, Japan; ^2^Department of Neurology, Saitama Sekishinkai Hospital, Saitama, Japan; ^3^Department of Neurosurgery, Saitama Sekishinkai Hospital, Saitama, Japan

## Abstract

We treated a patient with neurosarcoidosis, which caused the syndrome of inappropriate secretion of antidiuretic hormone (SIADH), in whom diagnosis was performed using neuroendoscopy. The patient was a 56-year-old female who was hospitalized for hyponatremia and diagnosed with SIADH based on a detailed examination. During the course, she developed impaired consciousness due to acute hydrocephalus, which improved after ventricular drainage. Head magnetic resonance imaging (MRI) confirmed nodular lesions at the floor of the third ventricle and the cerebral aqueduct. Neuroendoscopic biopsy led to the diagnosis of neurosarcoidosis. Her hyponatremia improved after steroid therapy. Neurosarcoidosis can cause SIADH, and complication of hydrocephalus may lead to a poor prognosis. Neuroendoscopy appears to be effective for the diagnosis of neurosarcoidosis with hydrocephalus and helps in deciding the treatment modality.

## 1. Introduction

Sarcoidosis is a disease of unknown etiology, which results in the formation of systemic noncaseating epithelioid granulomatous disease. Although approximately 5% of sarcoidosis cases are accompanied by neural lesions [1], there are no specific blood tests or imaging findings for neurosarcoidosis. Therefore, histopathological testing must be performed to reach a definitive diagnosis, and many cases present with difficulties [2]. Neurosarcoidosis is also known to cause diabetes insipidus [1]. However, neurosarcoidosis-induced syndrome of inappropriate secretion of antidiuretic hormone (SIADH) has rarely been described [3]. In this report, we present a case of a patient with SIADH in whom neuroendoscopy was used to establish the diagnosis of neurosarcoidosis.

## 2. Case Presentation

**Figure 1:**
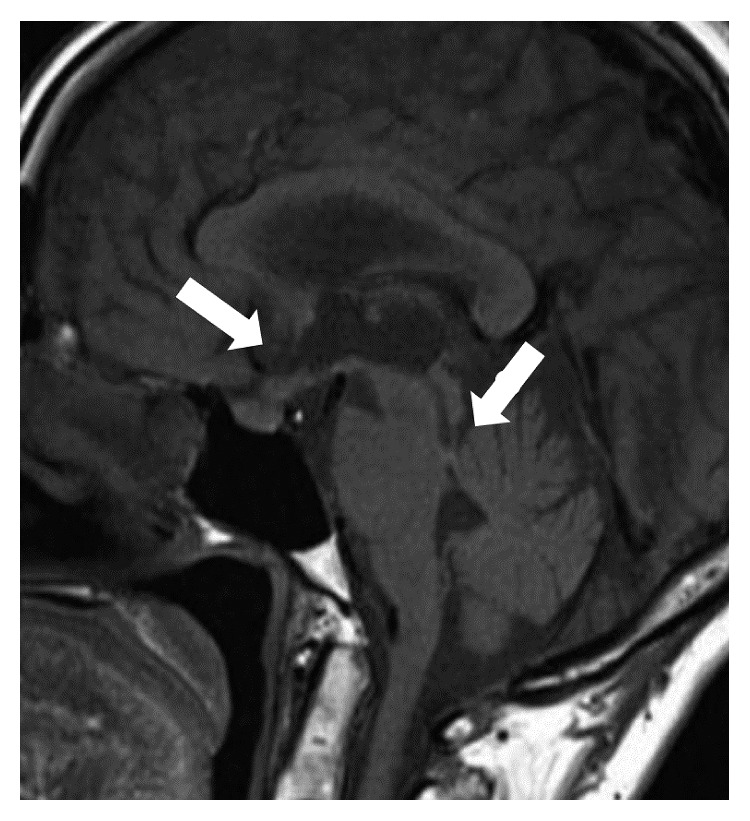
Head MRI showing thickening of the floor of the third ventricle and lesions in the arachnoid and pia membranes (arrows).

**Figure 2:**
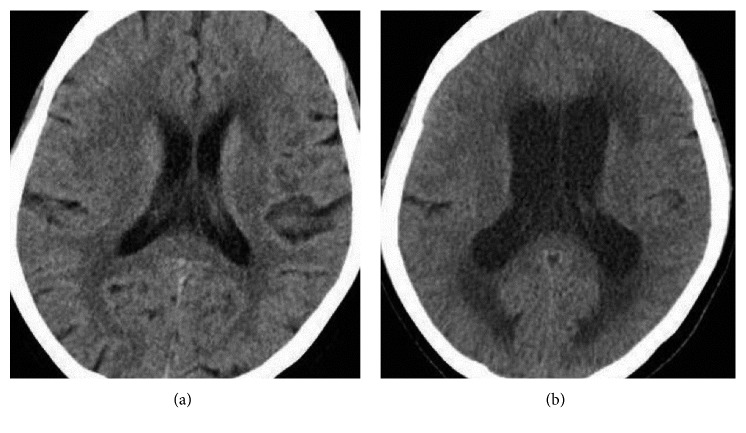
Head CT images at hospital admission (a) and on day 16 (b). The lateral ventricle on day 16 is larger than that at hospital admission.

**Figure 3:**
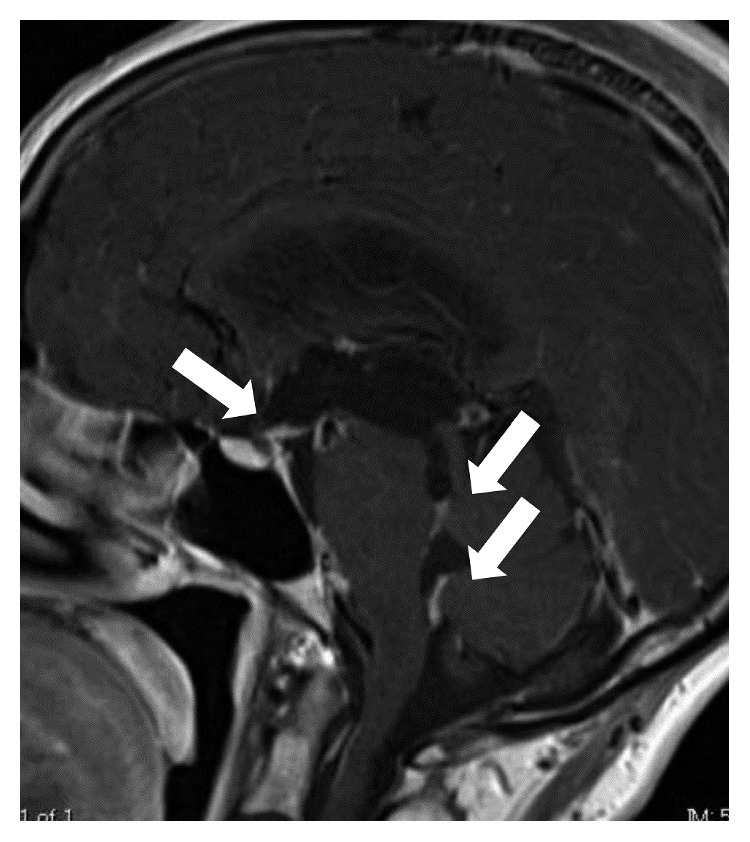
Head MRI showing contrast-enhanced nodular lesions on the floor of the third ventricle, aqueduct of the midbrain, and fourth ventricle.

**Figure 4:**
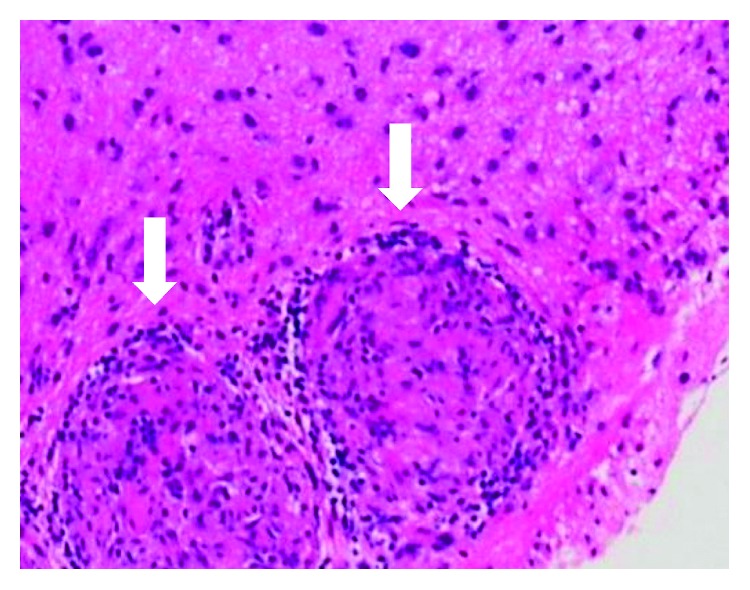
Hematoxylin and eosin-stained sections of nodular lesions on the floor of the third ventricle. Noncaseating epithelioid cell granulomas are observed (arrows).

**Figure 5:**
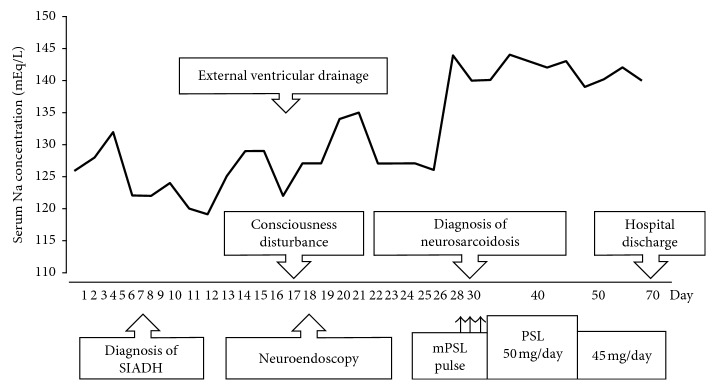
Clinical course from the day of hospital admission. The serum sodium level improved after initiation of steroid therapy. SIADH: syndrome of inappropriate secretion of antidiuretic hormone; mPSL: methylprednisolone; PSL: prednisolone; Na: sodium.

A 56-year-old female with no remarkable medical history presented to the emergency department with a chief complaint of dizziness since 3 days. Her blood tests revealed hyponatremia (serum Na^+^: 126 mEq/L), due to which she was hospitalized. There was no history of eating disorder, use of medications, or edema. Liver and renal functions were normal, and there was no metabolic abnormality, such as diabetes mellitus. Her serum osmotic pressure was low (254 mOsm/Kg), while urine osmolality was high (565 mOsm/Kg). Urinary Na^+^ levels were elevated (206 mEq/L). The adrenal gland and thyroid function were normal, while plasma ADH secretion was elevated (2.8 pg/mL), which led to the diagnosis of SIADH. Head MRI for the evaluation of central nervous system disease showed thickening of the floor of the third ventricle and lesions in the arachnoid and pia mater. Thus, neurosarcoidosis was suspected (Figure 1); however, levels of serum angiotensin-converting enzyme and soluble interleukin-2 receptor were not elevated. Moreover, there were no typical lesions that indicated sarcoidosis, such as rash, uveitis, or hilar lymphadenopathy. On day 16, the patient suddenly exhibited impaired consciousness; head computed tomography (CT) showed ventricular enlargement, and she was therefore diagnosed with acute hydrocephalus (Figure 2). Serum Na^+^ levels were low (122 mEq/L); however, there was no rapid progress of hyponatremia, and the cause of impaired consciousness was assumed to be acute hydrocephalus. Ventricular drainage led to improved consciousness, and contrast-enhanced head MRI confirmed nodular lesions with contrast effects in the floor of the third ventricle, cerebral aqueduct, and fourth ventricle (Figure 3). On day 18, neuroendoscopic fenestration of the floor of the third ventricle was performed, and biopsy specimen of nodular lesions was obtained. Histopathological examination showed noncaseating epithelioid cell granulomas (Figure 4). As there were no other lesions indicative of sarcoidosis, the diagnosis of sporadic neurosarcoidosis was made. Steroid therapy was initiated on day 26, and serum Na^+^ levels were restored to normal. Her symptoms did not exacerbate after gradual reduction in the dose of steroid. On day 70 of the illness, the patient was discharged on the basis of her independent gait (Figure 5).

## 3. Discussion

The present case illustrated that neurosarcoidosis can cause SIADH and that neuroendoscopy is a valuable aid for the diagnosis and treatment of neurosarcoidosis.

Sarcoidosis is a systemic granulomatous disease of unknown etiology; neuropathy occurs in approximately 5% of all cases [1]. The formation of granulomas in the hypothalamic-pituitary system impairs ADH secretion and transport pathways [4, 5]. Therefore, central diabetes insipidus complication occurs in 1%-2% of cases of sarcoidosis; however, cases that develop SIADH due to sarcoidosis are rare [3]. There are reports of systemic vasculitis-induced stimulation of the hypothalamic-pituitary system, which promotes ADH secretion and leads to the development of SIADH [6]; hydrocephalus-induced impaired osmotic regulation as the underlying mechanism for the development of SIADH has also been described [7]. Thus, it can be speculated that these mechanisms may have caused SIADH in the present case. As serum Na^+^ levels rapidly improved after steroid therapy instead of ventricular drainage, it was assumed that inflammation due to neurosarcoidosis stimulated the hypothalamic-pituitary system and promoted ADH secretion, which led to the development of SIADH.

Diagnosis of neurosarcoidosis requires histopathological examination because there are no specific blood tests or imaging modalities; thus, the diagnosis is often challenging [2]. Furthermore, neurosarcoidosis with hydrocephalus has a poor prognosis, and the mortality rate is high [8]. Hydrocephalus is caused by blockage of the cerebral aqueduct, which in turn is caused by disordered spinal fluid absorption due to sarcoidosis lesions or spread of granuloma lesions and inflammation [9]. With neuroendoscopy, the mechanism of development of hydrocephalus can be confirmed, and neuroendoscopic ventriculostomy can be performed in patients with obstructive hydrocephalus [10]. Furthermore, tissue biopsy may lead to the definitive diagnosis of neurosarcoidosis [11, 12]. It could lead to appropriate use of steroids and immunosuppressants; thus, neuroendoscopy is useful for the diagnosis and treatment of neurosarcoidosis.

In addition to diabetes insipidus, neurosarcoidosis may cause SIADH. Prognosis is poor in patients who develop a complication of hydrocephalus; neuroendoscopy is useful for the diagnosis of such cases and facilitates treatment decision-making.
